# Exploring the Microbiome of Diabetic Foot Ulcers: A Focus on Cases with a Clinical Worse Outcome

**DOI:** 10.3390/antibiotics14070724

**Published:** 2025-07-18

**Authors:** Laura Soldevila-Boixader, Anna Carrera-Salinas, Isabel Mur, Laura Morata, Alba Rivera, Jordi Bosch, Abelardo Montero-Saez, Jéssica Martínez Castillejo, Natividad Benito, Sara Martí, Oscar Murillo

**Affiliations:** 1 Infectious Diseases Unit, Department of Infectious Diseases, Bellvitge University Hospital, The Bellvitge Biomedical Research Institute (IDIBELL), University of Barcelona (UB), L’Hospitalet de Llobregat, 08907 Barcelona, Spain; 2 Internal Medicine, Infectious Diseases Unit, Hospital Moisès Broggi, 08970 Sant Joan Despí, Spain; 3 Study Group on Osteoarticular Infections of the Spanish Society of Clinical Microbiology and Infectious Diseases (GEIO-SEIMC), 28003 Madrid, Spain; 4 Department of Microbiology, Bellvitge University Hospital, The Bellvitge Biomedical Research Institute (IDIBELL), University of Barcelona (UB), L’Hospitalet de Llobregat, 08907 Barcelona, Spain; 5 Infectious Diseases Unit, Department of Medicine, Hospital de la Santa Creu i Sant Pau Hospital, Institut de Recerca Sant Pau (IR SANT PAU), Universitat Autonoma de Barcelona (UAB), 08041 Barcelona, Spain; 6 Department of Infectious Diseases, Clinic Hospital Barcelona, August Pi i Sunyer Biomedical Research Institute (IDIBAPS), University of Barcelona (UB), 08036 Barcelona, Spain; 7 Department of Microbiology, Hospital de la Santa Creu i Sant Pau, Institut de Recerca Sant Pau (IR SANT PAU), 08041 Barcelona, Spain; 8 Department of Microbiology, Clinic Hospital, ISGlobal, 08036 Barcelona, Spain; 9 Internal Medicine Department, Bellvitge University Hospital, The Bellvitge Biomedical Research Institute (IDIBELL), University of Barcelona (UB), L’Hospitalet de Llobregat, 08907 Barcelona, Spain; 10 Department of Nursery in Orthopedic Surgery, Clinic Hospital, 08036 Barcelona, Spain; jesmarti@clinic.cat; 11 The University of Queensland Center for Clinical Research (UQCCR), Brisbane 4029, Australia; 12 Centro de Investigación Biomédica en Red en Enfermedades Infecciosas (CIBERINFEC), National Institute of Health Carlos III (ISCIII), 28029 Madrid, Spain; 13 Research Network for Respiratory Diseases (CIBERES), National Institute of Health Carlos III (ISCIII), 28029 Madrid, Spain

**Keywords:** microbiome, chronic diabetic foot ulcers, Gammaproteobacteria

## Abstract

**Background/Objectives**: We evaluated the diabetic foot ulcer (DFU) microbiome in clinical situations identified as risk factors for a worse outcome and explored the roles of the most abundant microorganisms. **Methods**: A prospective multicenter cohort of diabetic patients with DFU were followed up for 6 months. We obtained a DFU tissue biopsy for microbiome analysis at the baseline visit. Genomic DNA was extracted (QIAamp DNA Mini Kit, Qiagen, Hilden, Germany) and quantified (QuantiFluor dsDNA System, Promega, Madison, WI, USA), with analysis of bacterial communities focusing on relative abundances (RA) and on alpha and beta diversity. **Results**: Overall, 59 DFUs were analyzed. DFUs of long duration (≥4 weeks) presented a higher RA of Gammaproteobacteria compared with ulcers of short duration (*p* = 0.02). Non-infected DFUs had a higher proportion of Actinobacteriota phyla than infected DFUs and, particularly, a higher RA of *Corynebacterium* genera (means ± SD: 0.063 ± 0.14 vs. 0.028 ± 0.13, respectively; *p* = 0.03). Regarding the pathogenic role of *Staphylococcus aureus*, DFUs with low *S. aureus* bacterial loads (<10^6^ CFU/mL) compared with those with high loads (≥10^6^ CFU/mL) showed a higher *Corynebacterium* RA (0.045 ± 0.08 vs. 0.003 ± 0.01, respectively; *p* = 0.01). **Conclusions**: In clinical situations associated with poor DFU outcomes, we observed a predominance of Gammaproteobacteria in the microbiome of long-duration ulcers and a higher RA of *Corynebacterium* in non-infected DFUs. An inverse relationship between the predominance of *Corynebacterium* and the *S. aureus* bacterial load in DFUs was also noted, which may suggest these commensals have a modulatory role. Further studies should explore the clinical utility of microbiome analysis for DFUs.

## 1. Introduction

Diabetic foot represents a major global health concern, primarily resulting from diabetes-related peripheral neuropathy and vasculopathy. These complications compromise nerve function and blood supply in the lower extremities, frequently leading to the development of foot ulcers. Diabetic foot ulcers (DFUs) are very common in patients with diabetes; between 15% and 25% of diabetic patients are expected to develop a DFU during their lifetime, and they become infected over time in approximately 40% of cases and are associated with high morbidity and hospitalization [1,2]. Several clinical risk factors are linked to a worse prognosis as DFUs evolve, among which the duration of the DFU and the presence of ischemia and infection are of particular relevance [3,4].

The DFU microbiome refers to the polymicrobial matrix of microorganisms within ulcers, which establish relationships between them and can impair wound healing. The study of this microbiome is complex and ongoing, but the recent use of molecular techniques to characterize bacteria genetically represents a novel improvement over existing approaches. The presence of particular microorganisms considered virulent and pathogenic within DFUs, such as Gram-negative bacilli (GNB) or *Staphylococcus aureus*, has been associated with a worse DFU prognosis [3,5,6]. On the other hand, commensal microorganisms are part of the skin’s normal flora, such as coagulase-negative staphylococci, and may help maintain microbial balance. Altogether, there is limited knowledge about the role of commensals and the relationships between commensal and pathogenic bacteria within DFUs [7,8].

Also, few works have provided knowledge that allow the establishment of a definitive link between clinical situations and different microbiome compositions. Overall, a more comprehensive understanding of the DFU bioburden, including the microbial relationships and microbiome composition, could improve our knowledge of DFU outcomes.

The clinical characteristics and outcomes of a multicenter cohort of patients with diabetes and DFUs have been reported previously [9]. In the present study using this cohort, our main aim was to evaluate the DFU microbiome from patients in clinical situations identified as risk factors for worse outcomes. We also aimed to explore the role of specific and abundant microorganisms in DFUs, as well as their relationships with *S. aureus*, a frequent cause of infection in DFUs.

## 2. Results

Overall, 59 DFU tissue samples were obtained at the baseline visits of 65 patients. Table 1 summarizes the baseline demographic and clinical characteristics of patients with DFU (*n* = 59).

The microbiome composition, including the most relevant taxa identified at different taxonomic levels, is analyzed. Overall, the most abundant phyla, class, and genera were Proteobacteria and Firmicutes, Alphaproteobacteria and Bacilli, and *Paracoccus* and *Staphylococcus*, respectively.

**Figure 1:**
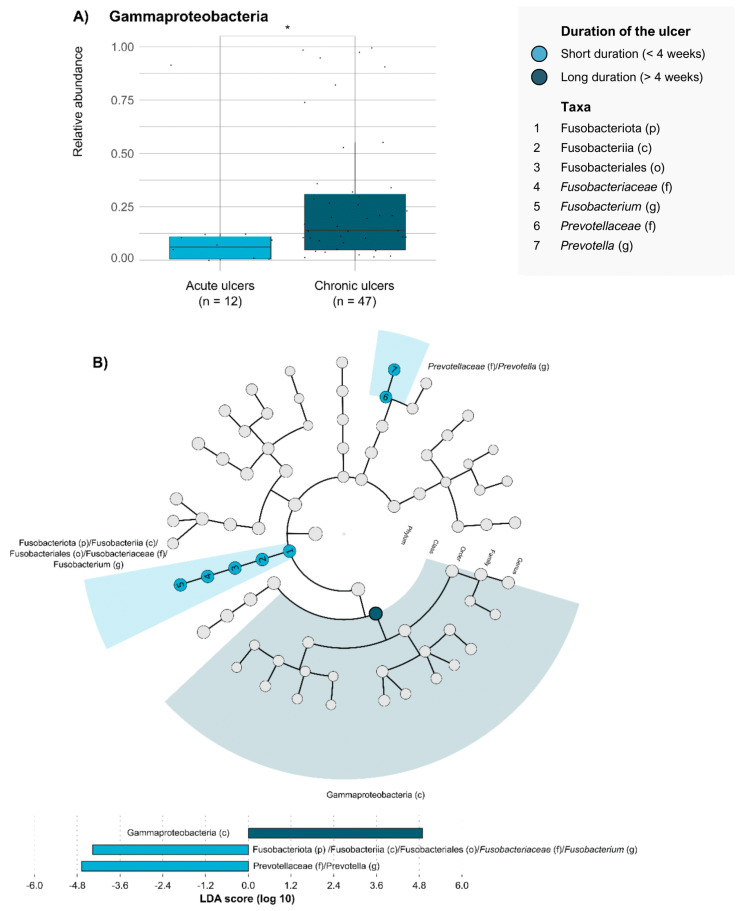
**Taxonomic groups that differentiate between DFUs with short and long duration**. (**A**) Comparison of relative abundance of Gammaproteobacteria between DFUs with short and long duration. Each dot represents the relative abundance of Gammaproteobacteria observed in a DFU sample. Each box plot shows the median, upper, and lower quartiles (boxes) and the 1.5 × IQR (inter-quartile range) values (vertical lines) for each group. Statistical comparison was conducted using the Mann–Whitney–Wilcoxon test. * *p*-value < 0.05. (**B**) Differentially enriched taxonomic groups in the microbiota of DFUs with short and long duration based on the linear discriminant analysis (LDA) effect size (LEfSE) (LDA score > 4). p, phylum; c, class; o, order; f, family; g, genus.

When analyzing the microbiome of DFUs in relation to clinical factors associated with poor prognosis, we observed significant compositional differences based on both ulcer duration and the presence of infection (Figure S1A). Thus, the Gammaproteobacteria class, which includes most pathogenic fermentative and non-fermentative GNB, showed higher mean RAs in DFUs of long duration (≥4 weeks; *n* = 47; 0.2655 ± 0.3032) compared with those of short duration (<4 weeks; *n* = 12; 0.1253 ± 0.2538; *p*-value = 0.02; Figure 1A). However, this predominance of Gammaproteobacteria was not associated with an increase in any of the underlying orders, families, or genera (Figure 1B). By contrast, compared to DFUs of long duration, those of short duration showed higher mean RAs of *Fusobacterium* (0.0095 ± 0.0429) and *Prevotella* (0.0106 ± 0.0811) genera, although overall levels were very low in both cases.

**Figure 2:**
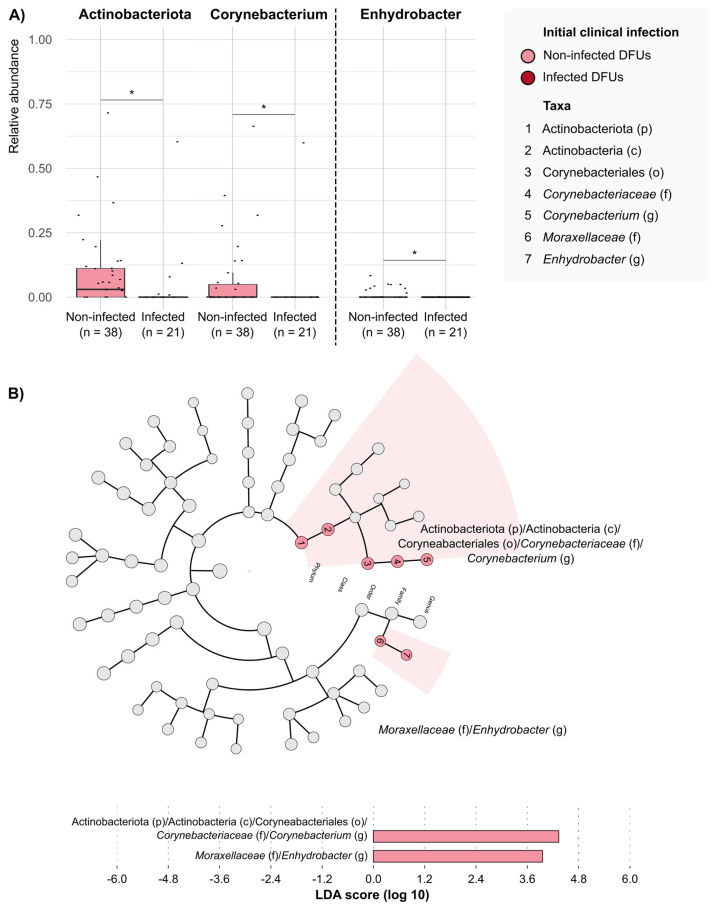
**Taxonomic groups that differentiate between infected and non-infected DFUs.** (**A**) Comparison of the relative abundances of taxonomic groups with significative differences between DFUs with and without initial clinical infection. Each dot represents the relative abundance observed in a DFU sample. Each box plot shows the median, upper, and lower quartiles (boxes) and the 1.5 × IQR (inter-quartile range) values (vertical lines) for each group. Statistical comparison was conducted using Mann–Whitney–Wilcoxon test. * *p*-value < 0.05. (**B**) Differentially enriched taxonomic groups in the microbiota of infected and non-infected DFUs based on the linear discriminant analysis (LDA) effect size (LEfSE) (LDA score > 4). p, phylum; c, class; o, order; f, family; g, genus.

As for the presence of DFU infection (Figure 2), Actinobacteriota phyla had a significantly higher mean RA in non-infected DFUs (*n* = 38; 0.0909 ± 0.1519) than in infected DFUs (0.0397 ± 0.1330; *p*-value = 0.017) (Figure S1B). Of note, we observed a higher mean RA of *Corynebacterium* genera among non-infected DFUs compared with infected DFUs in this phylum (0.0632 ± 0.1392 vs. 0.0285 ± 0.1306, respectively; *p*-value = 0.03).

We found no significant differences in microbiome composition by the presence of other relevant risk factors, such as ischemia, ulcer location, and prior antibiotic therapy. However, the DFU samples of patients with high glycosylated hemoglobin values (≥7%) presented higher mean RAs of the Alphaproteobacteria class compared to those with low glycosylated hemoglobin values (<7%; *p* = 0.024). The microbiome in cases with low glycosylated hemoglobin values showed a predominance of the Gammaproteobacteria class (*p* = 0.04).

**Figure 3:**
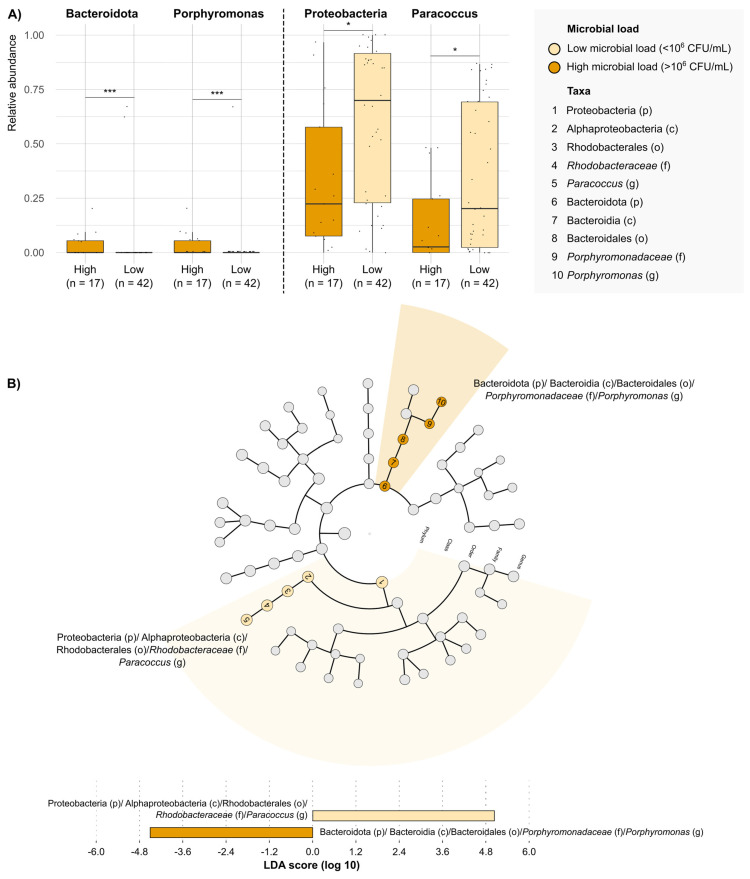
**Taxonomic groups that differentiate between DFUs with high (≥10^6^ CFU/mL) and low (<10^6^ CFU/mL) microbial loads.** (**A**) Comparison of the relative abundances of taxonomic groups with significative differences between DFUs with high and low microbial loads. Each dot represents the relative abundance observed in a DFU sample. Each box plot shows the median, upper, and lower quartiles (boxes) and the 1.5 × IQR (inter-quartile range) values (vertical lines) for each group. Statistical comparison was conducted using Mann–Whitney–Wilcoxon test. * *p*-value < 0.05, *** *p*-value < 0.001. (**B**) Differentially enriched taxonomic groups in the microbiota of DFUs with high and low microbial loads based on the linear discriminant analysis (LDA) effect size (LEfSE) (LDA score > 4). p, phylum; c, class; o, order; f, family; g, genus.

Regarding the influence of microbiome composition on DFUs, we compared the microbiome profiles with the bacterial load obtained through quantitative microbiological cultures (Figure S1C). The comparison of microbiome diversity between DFU samples with a high (≥10^6^ CFU/mL) and low (<10^6^ CFU/mL) microbial load is analyzed. The samples with high bacterial loads had statistically significant greater richness and alpha and beta diversities (*p* = 0.015). The differences between these groups in the RAs of various taxa from the level of phylum to genus are shown in Figure 3. DFUs with high microbial loads had higher mean RAs of the phylum Bacteroidota (0.0323 ± 0.0551 vs. 0.0308 ± 0.1396; *p*-value = 0.004); this was particularly associated with an increased RA of Porphyromonas when compared to DFUs with low bacterial loads (*p*-value = 0.001). By contrast, DFUs with low microbial loads presented a higher mean RA of the phylum Proteobacteria compared with high microbial loads (*p*-value = 0.017). Within this phylum, the most abundant class was Alphaproteobacteria, which included a diverse group of phototrophic and non-pathogenic GNB, with *Paracoccus* being the most frequent genera.

**Figure 4:**
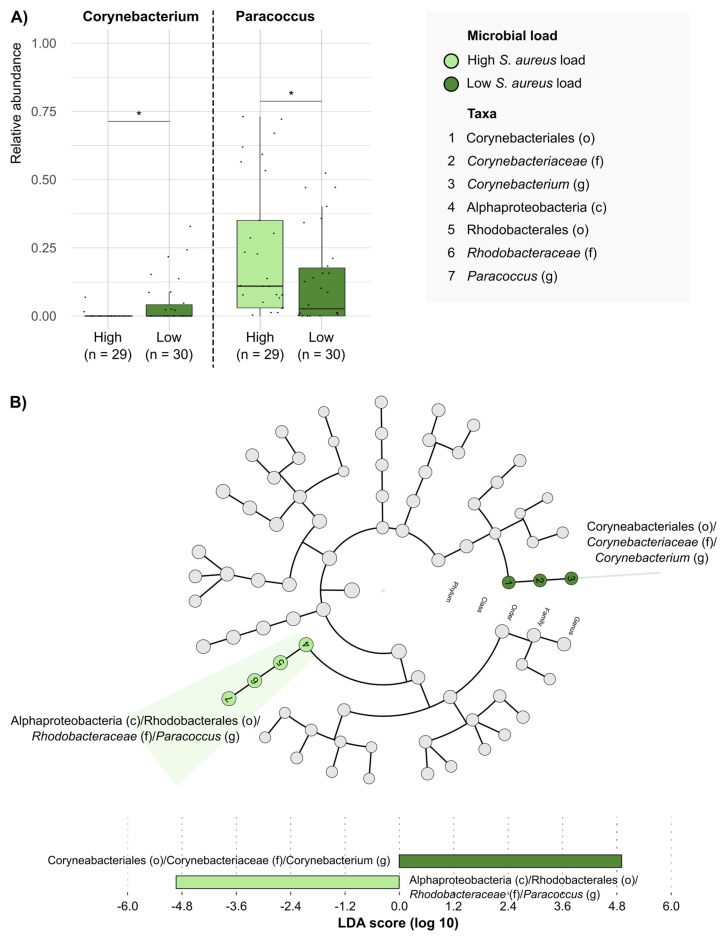
**Taxonomic groups that differentiate between DFUs with high and low *S. aureus* loads.** (**A**) Comparison of the relative abundances of taxonomic groups with significative differences between DFUs with high and low *S. aureus* loads. Each dot represents the relative abundance observed in a DFU sample. Each box plot shows the median, upper, and lower quartiles (boxes) and the 1.5 × IQR (inter-quartile range) values (vertical lines) for each group. Statistical comparison was conducted using Mann–Whitney–Wilcoxon test. * *p*-value < 0.05. (**B**) Differentially enriched taxonomic groups in the microbiota of DFUs with high and low microbial *S. aureus* loads based on the linear discriminant analysis (LDA) effect size (LEfSE) (LDA score > 4). c, class; o, order; f, family; g, genus.

In particular, we looked at the *S. aureus* load in DFUs because this is the most frequent causative agent in DFU infections (Figure 4). We observed that the microbiome from DFUs with a low *S. aureus* load (<10^6^ CFU/mL) presented a higher mean RA of the Corynebacteriales class and *Corynebacterium* genus than those with high *S. aureus* loads (0.0456 ± 0.0856 vs. 0.003 ± 0.0131; *p* = 0.0104).

## 3. Discussion

Our evaluation of the DFU microbiome of a cohort of patients with diabetes who attended three referral centers revealed significant differences in microbiome composition. This was notable in the DFUs of patients with some clinical conditions related to worse outcomes, such as a long DFU duration, the presence of infection, and the presence of high bacterial loads. We also observed the modulatory role of certain commensals on other microorganisms present in the microbiome. Together, these results could be used to inform advancements in the management of DFUs.

The DFU microbiome is a dynamic and organized polymicrobial community that can not only be influenced by external factors but also interfere with the evolution of DFUs [10,11]. Of interest, this bioburden is the niche for relevant interactions between microorganisms, with each bacterium having the potential to synergize with or downregulate the pathogenic role of others [12,13]. Our study has highlighted the complex and diverse composition of the DFU microbiome, including the high number of taxa. Indeed, the most abundant phyla were Proteobacteria, Firmicutes, and Actinobacteroidota, which represent the most common Gram-negative and Gram-positive pathogens and commensals. This complexity in the microbiome was particularly notable in DFUs with high bacterial loads, which showed statistically significant higher richness and alpha and beta diversities compared with low bacterial loads. Whereas the polymicrobial nature of the DFU microbiota has been well described, the bacterial diversity in these ulcers is difficult to study with traditional culture-based methods because these tend to over-represent microorganisms that grow easily in laboratory conditions. Molecular studies may therefore offer a more comprehensive approach to their investigation. Previous works using these new methods have shown that bacterial diversity is higher in ulcers than in healthy skin [14] and in ulcers of longer duration [15]. Based on our results, the higher diversity in DFUs was associated with the presence of high bacterial loads, the latter having been related to impaired ulcer healing and worse DFU outcomes in previous research [9,16,17]

Beyond the polymicrobial nature and bacterial diversity of the DFU microbiome, the role of particular bacteria or a group of microorganisms remains difficult to determine. Previously, the predominance of GNB in traditional cultures and ulcer duration of longer than 30 days have been identified as risk factors for worse DFU outcomes [10], and the analysis of the DFU microbiome can help clarify the impact of differences in its composition. As an example, the phylum Proteobacteria includes Gammaproteobacteria and Alphaproteobacteria classes, which grouped different pathogenic and non-pathogenic GNB. Specifically, in comparison with short-duration DFUs, we observed that the DFU microbiome in ulcers with long durations had higher mean RAs of the Gammaproteobacteria class that includes most relevant pathogenic GNB. These results seem consistent with previous works; in the clinical setting, Gardner et al. analyzed the microbiome of non-ischemic DFUs and reported a positive correlation between the RA of Proteobacteria and the duration of those ulcers, but reported a negative correlation with the abundance of *Staphylococcus* [18]. Using an animal model of DFUs, other authors have shown a progressive shift in bacterial predominance from Firmicutes to Proteobacteria associated with non-healing ulcers. By contrast, the predominance of the Alphaproteobacteria class, which includes mostly non-pathogenic GNB, was not associated with any duration of DFU. An important group of anaerobic GNB, such as the *Fusobacterium* genera, had a higher RA in DFU microbiomes of short durations compared with those of long durations. Thus, given this previous information about the association between GNB abundance, long DFU duration, and worse DFU outcome, our results reinforce the role of Gammaproteobacteria from the Proteobacteria phylum in clinical scenarios of non-healing DFUs whether the prognosis is worse. The increased abundance of Gammaproteobacteria in long-duration DFUs may have relevant clinical implications. This class includes pathogens like *Pseudomonas aeruginosa* and *Enterobacter* spp., commonly linked to chronic infection and antibiotic resistance. Their presence may be associated with impaired wound healing, supporting earlier use of broad-spectrum antibiotics or surgical intervention in selected patients.

To our knowledge, poor control of diabetes mellitus has been associated with an increased risk of specific complications (i.e., vascular disease), but a relationship between control and the prognosis of DFU has not been established. In our experience, we observed a great abundance of the Alphaproteobacteria class in DFUs from patients with high glycosylated hemoglobin values and a predominance of the Gammaproteobacteria class in cases with better diabetes disease control. It will be interesting to explore the possible relationship between the DFU microbiome and disease control in diabetes, just as the relationships of the human microbiome with other diseases have been investigated [19,20,21,22]. It is also important to consider the dynamic shifts in the wound microbiome, as highlighted in only a few studies to date. In one such study involving 28 patients with diabetic heel ulcers, serial biopsy samples were collected over time to evaluate microbial diversity and longitudinal changes. Healing ulcers exhibited greater microbial diversity and temporal variability, with microbial communities typically dominated by skin commensals and non-pathogenic taxa. In contrast, non-healing ulcers demonstrated persistent colonization by dysbiotic communities, particularly anaerobes and members of the Enterobacteriaceae family, which remained stable across multiple time points [23].

We note the high RA of Actinobacteriota phylum in non-infected DFUs compared with infected DFUs, and particularly, the predominance of the commensal genera *Corynebacterium*. The microbiota of our skin directly regulates cutaneous health and disease; particularly, these commensals interact with wound-repairing skin cells to enhance barrier regeneration [24], and some of them may interact with bacterial pathogens to modulate their virulence [12,25]. In this line, we observed an inverse association between *Corynebacterium* and *S. aureus*, with a high RA of *Corynebacterium* spp. in DFUs with low *S. aureus* loads; thus, our results suggest a potential modulating effect of *Corynebacterium* on *S. aureus* growth. Although *S. aureus* is the most frequent causative agent of DFU infection, its interaction with the human niche can change continuously from commensal to pathogen. Few previous works have reported that the presence of *Corynebacterium* in vitro decreased the transcription of virulence genes of *S. aureus* compared with *S aureus* cultures alone [25]. It will be interesting to evaluate the role of *Corynebacterium* and other commensals on DFUs further as this might help clinicians interpret their presence in conventional cultures.

This study has several limitations that warrant discussion. Our results were obtained in the outpatient departments of hospitals that receive referrals for the management of complex diabetic foot disease. Thus, the generalizability of our results may be limited in cases where patients with DFUs in other settings. In addition, the DFU microbiome analysis may have been affected by the presence of uncontrolled clinical factors, due in part, to existing limitations in our knowledge of this field. Finally, the small sample size precluded further analysis and comparison between subgroups, and no additional microbiome biopsy was performed at the 6-month follow-up. Despite these limitations, our study provides relevant results that may help in the management of DFUs.

All these findings from the microbiome study have potential clinical implications. The predominance of Gammaproteobacteria in long-duration DFUs may support the implementation of early and more aggressive therapeutic strategies, including empirical antibiotic treatment and close clinical monitoring.

Conversely, the increased relative abundance of *Corynebacterium* spp., particularly in non-infected DFUs with low *S. aureus* bacterial load, suggests that certain commensals could serve as biomarkers of lower infection risk. Previous studies have shown that *Corynebacterium* may downregulate *S. aureus* virulence gene expression, and our findings further support the need to explore protective microbial interactions in DFUs. Microbiome profiling could contribute to risk stratification and help guide personalized interventions, such as the modulation of the wound environment, potentially through targeted topical therapies.

## 4. Materials and Methods

### 4.1. Study Design, Setting, and Patients

A prospective multicenter cohort study was performed in the outpatient settings of three Spanish referral hospitals that managed diabetic foot disease in multidisciplinary teams. From September 2017 to August 2019, we included all patients with diabetes aged >18 years who presented with a DFU and were treated in these teams. Due to their different pathogenesis, we excluded ulcers on amputated stumps and that presented after surgery. These types of ulcers were excluded because their microbiological profile may differ from that of typical diabetic foot ulcers, as post-surgical and stump ulcers may reflect nosocomial colonization rather than community-acquired diabetic foot microbiota. The ethics committees of all three hospitals approved the present study (REF PR 135/17), and all patients signed an informed consent form before participating.

### 4.2. Definitions, Data Collection, and Clinical Management

We recorded the main clinical characteristics at the baseline visit and during follow-up. The main cohort characteristics and variable definitions, including the cutoffs for glycosylated hemoglobin (< or >7%) and high bacterial load (≥10^6^ UFC/mL), have been reported previously [9].

Of note, a DFU was defined as a foot ulcer due to diabetic neuropathy (neuropathic ulcer) with or without peripheral artery disease (neuro-ischemic ulcer), diagnosed based on clinical symptoms of intermittent claudication, rest pain, or abnormalities on non-invasive vascular assessment [26,27]. The duration of DFU was defined as long (≥4 weeks) or short (<4 weeks). DFU infection was diagnosed according to the clinical criteria proposed by the International Working Group on the Diabetic Foot [28] as the presence of at least two of the following criteria: erythema, local swelling or induration, local tenderness or pain, local warmth, or purulent discharge.

All included patients underwent a DFU tissue biopsy at the baseline visit, which was obtained and processed according to established methods [9]. The tissue sampling protocol was standardized across all participating centers.

The attending team diagnosed clinical infection and decided on the most appropriate antimicrobial therapy after obtaining the DFU tissue sample [29,30]. Other appropriate measures, including wound care, dressings, and offloading, were performed according to national guidelines. Patients were followed up for 6 months.

### 4.3. Microbiological Studies and Statistical Analyses

DFU tissue samples were processed according to established methods to obtain quantitative cultures, as previously described [9], and classified into high microbial load (≥10^6^ CFU/mL) or low microbial load (<10^6^ CFU/mL). The same tissue samples were also used for microbiome analysis. The study design and the resolution of the sequencing methodology used for microbiome analysis only allowed classification at the genus level, not at the species level. However, we identified samples with high *Staphylococcus aureus* loads (≥10^6^ CFU/mL) using quantitative culture methods. Genomic DNA was extracted using the QIAamp DNA Mini Kit (Qiagen, Hilden, Germany) and quantified by the QuantiFluor dsDNA System (Promega, Madison, WI, USA). The hypervariable V3 and V4 regions of the 16S rRNA gene were amplified using the primers 16S Amplicon 341F (5′-TCGTCGGCAGCGTCAGATGTGTATAAGA GACAGCCTACGGGNGGCWGCAG-3′) and 805R (5′-GTCTCGTGGGCTCGGAGATGTGTATAAGAGACAGGACTACHVGGGTATCTAATCC-3′). This region of the 16S rRNA gene was selected because it provides a high level of taxonomic resolution, helping to distinguish between closely related taxa. Following gene amplification, 1 µL of the libraries were run on a Bioanalyzer DNA 1000 chip to verify the size (~550 bp), followed by paired-end sequencing (2 × 300 bp) on a MiSeq platform (Illumina, San Diego, CA, USA). To minimize the influence of contaminants, PCR-negative controls were included in the sequencing process.

R software version 4.2.1 was used for data processing and sequence analysis. Briefly, DADA2 version 1.20 [31] was used to quality-filter, denoise, merge all pair read sequences and, remove chimeras. This approach was selected for its high-resolution inference of amplicon sequence variants (ASVs), which provide more accurate and reproducible taxonomic identification compared to traditional OUT-based clustering. DADA2 was also used for taxonomic assignments using the SILVA v138 database [31]. ASVs designated as Archaea, chloroplast, and mitochondria were excluded from the dataset along with ASVs lacking phylum and class assignment to avoid inclusion of non-bacterial or ambiguously classified sequences that could bias microbiome analysis.

The analysis of bacterial communities, including the relative abundance (RA) of each ASV, the alpha diversity and beta diversity, was performed using phyloseq R package [32]. Amplicon sequence variants (ASVs) designated as Archaea, chloroplast, and mitochondria were excluded from the dataset along with ASVs lacking phylum and class assignment.

The analysis of bacterial communities, including the relative abundance (RA) of each ASV and the alpha diversity (Chao1, Shannon, and Simpson indexes) and beta diversity (Bray–Curtis index), was performed using phyloseq R package [33] to determine the differences between variables of interest: duration of DFU, microbial load, diagnosis of DFU infection, and outcome at 6 months. Briefly, alpha diversity, used to assess within-sample diversity, was estimated using Chao1, Shannon and Simpson indexes. These complementary metrics were used to capture different aspects of microbial diversity, including richness, abundance distribution, and dominance. Homoscedasticity of the alpha diversity variance was calculated using Levene’s test. Statistical significance of alpha diversity was evaluated using the Mann–Whitney–Wilcoxon test (for pairwise comparisons) or the Kruskal–Wallis test (for multiple groups), as appropriate. Non-parametric tests were chosen due to the non-normal distribution of diversity indexes and the relatively small sample size.

Beta diversity (between-sample dissimilarity), was assessed by Bray–Curtis distance matrices to quantify differences in microbial community composition based on taxa abundance profiles. Beta diversity differences were tested using the adonis test (permutational multivariate analysis of variance) from the vegan R package, as it allows for robust comparison of multivariate dispersion among groups based on dissimilarity matrices.

RA analyses of the compositional data were filtered to remove the less prevalent ASVs (RA < 0.001) to minimize noise from rare taxa or contaminants. Group comparisons of RA were performed using the Mann–Whitney–Wilcoxon test or Kruskal–Wallis test, as appropriate. Linear discriminant analysis effect size (LEfSE) analysis was used to identify differentially enriched ASVs in each variable of interest [34], using a linear discriminant analysis score of >4 to ensure the biological relevance and robustness of the identified taxa. Heatmaps of microbial RA were generated using the ampvis2 R package. This tool was selected for its capacity to visualize complex microbial community patterns across metadata-defined groups (https://kasperskytte.github.io/ampvis2/articles/ampvis2.html). *p*-values < 0.05 were considered statistically significant.

## 5. Conclusions

In conclusion, we observed that DFUs in clinical situations associated with a worse prognosis had different microbiome compositions. Bacterial diversity was significantly higher in the microbiomes of DFU with high bacterial loads. Gammaproteobacteria, but no other class in the Proteobacteria phylum, were predominant in clinical scenarios of DFUs with long durations. The Actinobacteria class, including the commensal genera Corynebacterium, was more abundant in non-infected DFUs. Also, our results suggest that *Corynebacterium* had a modulating effect on *S. aureus* growth. These findings may have important implications for human health, particularly in the identification of microbial biomarkers that can aid in the early recognition of DFU severity and guide therapeutic decisions. The presence of Gammaproteobacteria may justify a more aggressive treatment approach in chronic DFUs, while *Corynebacterium* might represent a potential indicator of lower risk of *S. aureus* infection. Overall, our results meet the study objectives and highlight the potential role of the microbiome as a clinical tool to support clinicians in the management of DFUs.

## Figures and Tables

**Table 1:** Demographic and clinical characteristics.

*n = 59*	*Value*
Age (median, IQR)	65 years (59–71)
Male sex	47 patients (80%)
Type 2 diabetes mellitus (T2DM)	52 patients (88%)
BMI (median, IQR)	29 kg/m^2^ (25–33)
Years since diabetes diagnosis (median, IQR)	17 years (8.5–25)
Diabetic neuropathy	49 patients (83%)
Peripheral vasculopathy	30 (51%)
DFU duration ≥ 4 weeks	47 patients (80%)
Antibiotic use in the previous month	28 patients (48%)
DFU infection	21 (36%)

Data are presented as medians (IQR) or as number (%).

## Data Availability

The original contributions presented in this study are included in the article/Supplementary Materials. Further inquiries can be directed to the corresponding author.

## Supplementary Materials

The following supporting information can be downloaded at: https://www.mdpi.com/article/10.3390/antibiotics14070724/s1. Figure S1. Heatmaps showing the relative abundances of microbial taxa in diabetic foot ulcers (DFUs) categorized by clinical characteristics: (A) DFUs with short and long duration; (B) Infected and non-infected DFUs; (C) DFUs with high (≥10^6^ CFU/mL) and low (<10^6^ CFU/mL) microbial load.

## References

[1] Lazzarini P.A., Hurn S.E., Fernando M.E., Jen S.D., Kuys S.S., Kamp M.C., Reed L.F. (2015). Prevalence of Foot Disease and Risk Factors in General Inpatient Populations: A Systematic Review and Meta-Analysis. BMJ Open.

[2] Lavery L.A., Armstrong D.G., Wunderlich R.P., Mohler M.J., Wendel C.S., Lipsky B.A. (2006). Risk Factors for Foot Infections in Individuals With Diabetes. Diabetes Care.

[3] Lipsky B.A., Sheehan P., Armstrong D.G., Tice A.D., Polis A.B., Abramson M.A. (2007). Clinical Predictors of Treatment Failure for Diabetic Foot Infections: Data from a Prospective Trial. Int. Wound J..

[4] Uysal S., Arda B., Taşbakan M.I., Çetinkalp Ş., Şimşir I.Y., Öztürk A.M., Uysal A., Ertam İ. (2017). Risk Factors for Amputation in Patients with Diabetic Foot Infection: A Prospective Study. Int. Wound J..

[5] Kalan L.R., Meisel J.S., Loesche M.A., Horwinski J., Soaita I., Chen X., Uberoi A., Gardner S.E., Grice E.A. (2019). Strain- and Species-Level Variation in the Microbiome of Diabetic Wounds Is Associated with Clinical Outcomes and Therapeutic Efficacy. Cell Host Microbe.

[6] Sen P., Demirdal T., Emir B. (2019). Meta-Analysis of Risk Factors for Amputation in Diabetic Foot Infections. Diabetes Metab. Res. Rev..

[7] Macdonald K.E., Boeckh S., Stacey H.J., Jones J.D. (2021). The Microbiology of Diabetic Foot Infections: A Meta-Analysis. BMC Infect. Dis..

[8] MacDonald A., Brodell J.D., Daiss J.L., Schwarz E.M., Oh I. (2019). Evidence of Differential Microbiomes in Healing versus Non-Healing Diabetic Foot Ulcers Prior to and Following Foot Salvage Therapy. J. Orthop. Res..

[9] Soldevila-Boixader L., Mur I., Morata L., Sierra Y., Rivera A., Bosch J., Montero-Saez A., Fernández-Reinales A.J., Martí S., Benito N. (2022). Clinical Usefulness of Quantifying Microbial Load from Diabetic Foot Ulcers: A Multicenter Cohort Study. Diabetes Res. Clin. Pract..

[10] Liu C., Ponsero A.J., Armstrong D.G., Lipsky B.A., Hurwitz B.L. (2020). The Dynamic Wound Microbiome. BMC Med..

[11] Jneid J., Lavigne J.P., La Scola B., Cassir N. (2017). The Diabetic Foot Microbiota: A Review. Hum. Microb. J..

[12] Ngba Essebe C., Visvikis O., Fines-Guyon M., Vergne A., Cattoir V., Lecoustumier A., Lemichez E., Sotto A., Lavigne J.-P., Dunyach-Remy C. (2017). Decrease of *Staphylococcus aureus* Virulence by *Helcococcus kunzii* in a *Caenorhabditis elegans* Model. Front Cell. Infect. Microbiol..

[13] Stacy A., Everett J., Jorth P., Trivedi U., Rumbaugh K.P., Whiteley M. (2014). Bacterial Fight-and-Flight Responses Enhance Virulence in a Polymicrobial Infection. Proc. Natl. Acad. Sci. USA.

[14] Redel H., Gao Z., Li H., Alekseyenko A.V., Zhou Y., Perez-Perez G.I., Weinstock G., Sodergren E., Blaser M.J. (2013). Quantitation and Composition of Cutaneous Microbiota in Diabetic and Nondiabetic Men. J. Infect. Dis..

[15] Oates A., Bowling F.L., Boulton A.J.M., McBain A.J. (2012). Molecular and Culture-Based Assessment of the Microbial Diversity of Diabetic Chronic Foot Wounds and Contralateral Skin Sites. J. Clin. Microbiol..

[16] Xu L., McLennan S.V., Lo L., Natfaji A., Bolton T., Liu Y., Twigg S.M., Yue D.K. (2007). Bacterial Load Predicts Healing Rate in Neuropathic Diabetic Foot Ulcers. Diabetes Care.

[17] Gardner S.E., Frantz R.A. (2008). Wound Bioburden and Infection-Related Complications in Diabetic Foot Ulcers. Biol. Res. Nurs..

[18] Gardner S.E., Haleem A., Jao Y.L., Hillis S.L., Femino J.E., Phisitkul P., Heilmann K.P., Lehman S.M., Franciscus C.L. (2014). Cultures of Diabetic Foot Ulcers without Clinical Signs of Infection Do Not Predict Outcomes. Diabetes Care.

[19] Hou K., Wu Z.X., Chen X.Y., Wang J.Q., Zhang D., Xiao C., Zhu D., Koya J.B., Wei L., Li J. (2022). Microbiota in Health and Diseases. Signal Transduct. Target. Ther..

[20] Sanchez-Rodriguez E., Egea-Zorrilla A., Plaza-Díaz J., Aragón-Vela J., Muñoz-Quezada S., Tercedor-Sánchez L., Abadia-Molina F. (2020). The Gut Microbiota and Its Implication in the Development of Atherosclerosis and Related Cardiovascular Diseases. Nutrients.

[21] Hill J.M., Clement C., Pogue A.I., Bhattacharjee S., Zhao Y., Lukiw W.J. (2014). Pathogenic Microbes, the Microbiome, and Alzheimer’s Disease (AD). Front. Aging Neurosci..

[22] Farrell J.J., Zhang L., Zhou H., Chia D., Elashoff D., Akin D., Paster B.J., Joshipura K., Wong D.T.W. (2012). Variations of Oral Microbiota Are Associated with Pancreatic Diseases Including Pancreatic Cancer. Gut.

[23] Sloan T.J., Turton J.C., Tyson J., Musgrove A., Fleming V.M., Lister M.M., Loose M.W., Sockett R.E., Diggle M., Game F.L. (2019). Examining Diabetic Heel Ulcers through an Ecological Lens: Microbial Community Dynamics Associated with Healing and Infection. J. Med. Microbiol..

[24] Patel B.K., Patel K.H., Huang R.Y., Lee C.N., Moochhala S.M. (2022). The Gut-Skin Microbiota Axis and Its Role in Diabetic Wound Healing—A Review Based on Current Literature. Int. J. Mol. Sci..

[25] Ramsey M.M., Freire M.O., Gabrilska R.A., Rumbaugh K.P., Lemon K.P. (2016). *Staphylococcus aureus* Shifts toward Commensalism in Response to *Corynebacterium* Species. Front. Microbiol..

[26] Hinchliffe R.J., Brownrigg J.R.W., Apelqvist J., Boyko E.J., Fitridge R., Mills J.L., Reekers J., Shearman C.P., Zierler R.E., Schaper N.C. (2016). IWGDF Guidance on the Diagnosis, Prognosis and Management of Peripheral Artery Disease in Patients with Foot Ulcers in Diabetes. Diabetes Metab. Res. Rev..

[27] Armstrong D.G., Boulton A.J.M., Bus S.A. (2017). Diabetic Foot Ulcers and Their Recurrence. New Engl. J. Med..

[28] Bus S.A., Lavery L.A., Monteiro-Soares M., Rasmussen A., Raspovic A., Sacco I.C.N., van Netten J.J. (2019). IWGDF Guideline on the Prevention of Foot Ulcers in Persons with Diabetes. IWGDF Guidel..

[29] Lipsky B.A., Berendt A.R., Cornia P.B., Pile J.C., Peters E.J.G., Armstrong D.G., Deery H.G., Embil J.M., Joseph W.S., Karchmer A.W. (2012). 2012 Infectious Diseases Society of America Clinical Practice Guideline for the Diagnosis and Treatment of Diabetic Foot Infections. Clin. Infect. Dis..

[30] Lipsky B.A., Senneville É., Abbas Z.G., Aragón-Sánchez J., Diggle M., Embil J.M., Kono S., Lavery L.A., Malone M., van Asten S.A. (2020). Guidelines on the Diagnosis and Treatment of Foot Infection in Persons with Diabetes (IWGDF 2019 Update). Diabetes Metab. Res. Rev..

[31] Callahan B.J., McMurdie P.J., Rosen M.J., Han A.W., Johnson A.J.A., Holmes S.P. (2016). DADA2: High-Resolution Sample Inference from Illumina Amplicon Data. Nat. Methods.

[32] Quast C., Pruesse E., Yilmaz P., Gerken J., Schweer T., Yarza P., Peplies J., Glöckner F.O. (2012). The SILVA Ribosomal RNA Gene Database Project: Improved Data Processing and Web-Based Tools. Nucleic. Acids Res..

[33] McMurdie P.J., Holmes S. (2013). Phyloseq: An R Package for Reproducible Interactive Analysis and Graphics of Microbiome Census Data. PLoS ONE.

[34] Segata N., Izard J., Waldron L., Gevers D., Miropolsky L., Garrett W.S., Huttenhower C. (2011). Metagenomic Biomarker Discovery and Explanation. Genome Biol..

