# Supplementary material for: Exploring the Microbiome of Diabetic Foot Ulcers: A Focus on Cases with a Clinical Worse Outcome
Source: Antibiotics (Basel). 2025 Jul 18;14(7):724. doi: 10.3390/antibiotics14070724 (PMC12291833; doi:10.3390/antibiotics14070724)
Supplement: Supplementary file 1 [file antibiotics-14-00724-s001.zip › antibiotics-3703559-supplementary.pdf]

Supplementary Figure S1. Heatmaps showing the relative abundances of microbial taxa in diabetic foot ulcers (DFUs) categorized by clinical characteristics: A) DFUs with short and long duration; B) Infected and non-infected DFUs; C) DFUs with high ($\geq 10^6$ CFU/mL) and low ($<10^6$ CFU/mL) microbial load.

A)

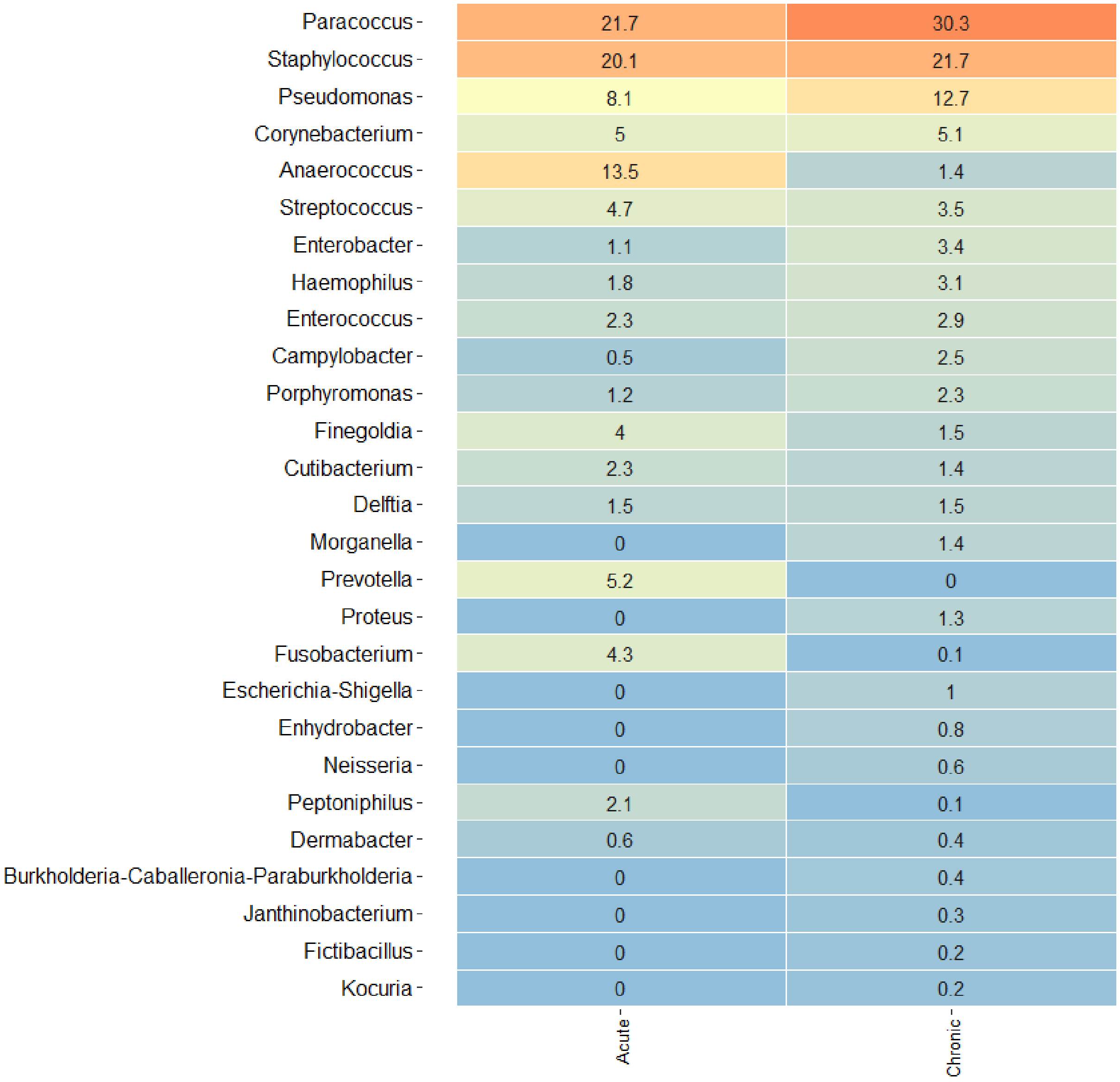

B)

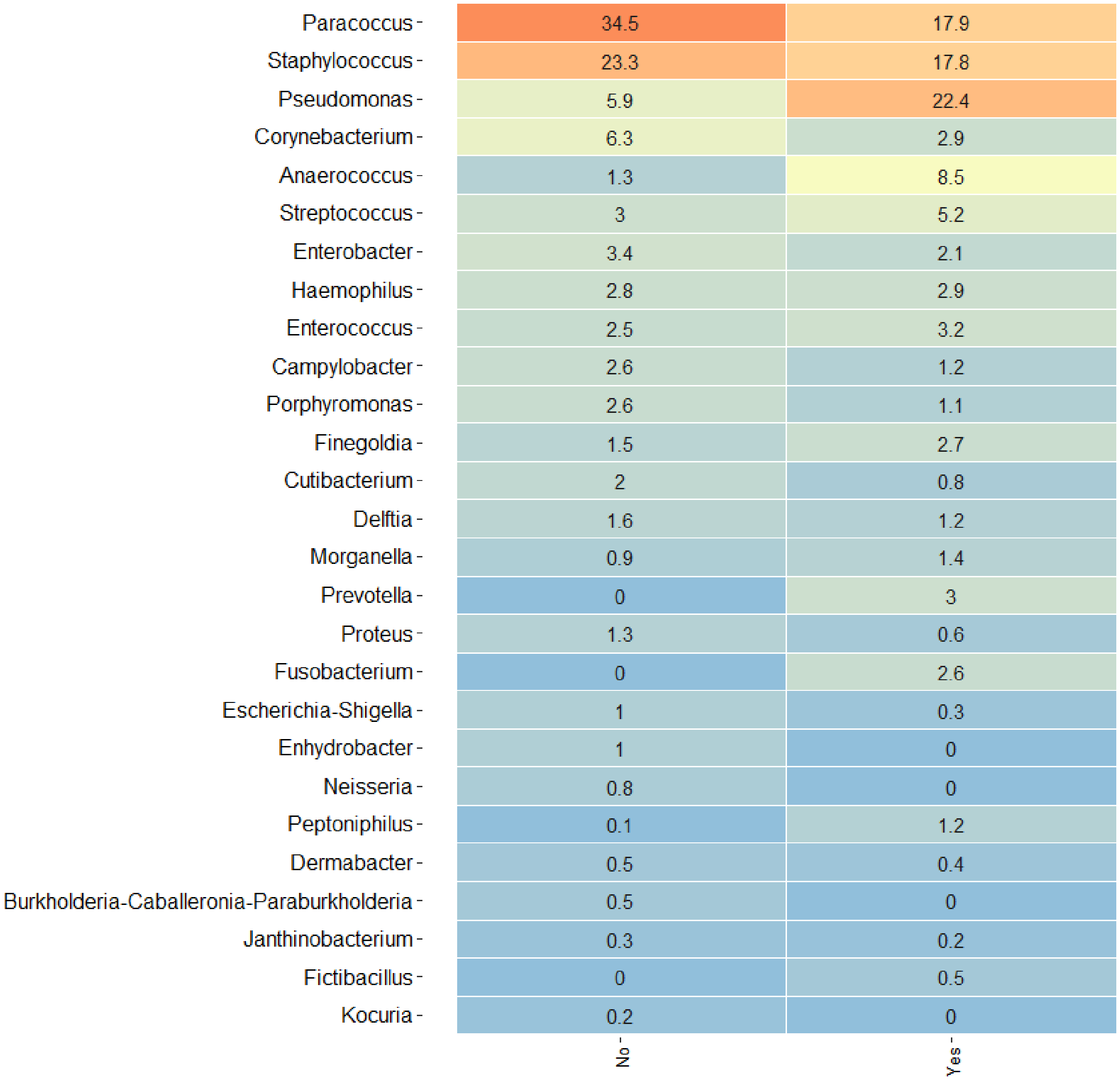
Paracoccus 34.5 17.9
Staphylococcus 23.3 17.8
Pseudomonas 5.9 22.4
Corynebacterium 6.3 2.9
Anaerococcus 1.3 8.5
Streptococcus 3 5.2
Enterobacter 3.4 2.1
Haemophilus 2.8 2.9
Enterococcus 2.5 3.2
Campylobacter 2.6 1.2
Porphyromonas 2.6 1.1
Finegoldia 1.5 2.7
Cutibacterium 2 0.8
Delftia 1.6 1.2
Morganella 0.9 1.4
Prevotella 0 3
Proteus 1.3 0.6
Fusobacterium 0 2.6
Escherichia-Shigella 1 0.3
Enhydrobacter 1 0
Neisseria 0.8 0
Peptoniphilus 0.1 1.2
Dermabacter 0.5 0.4
Burkholderia-Caballeronia-Paraburkholderia 0.5 0
Janthinobacterium 0.3 0.2
Fictibacillus 0 0.5
Kocuria 0.2 0
No
Yes

C)

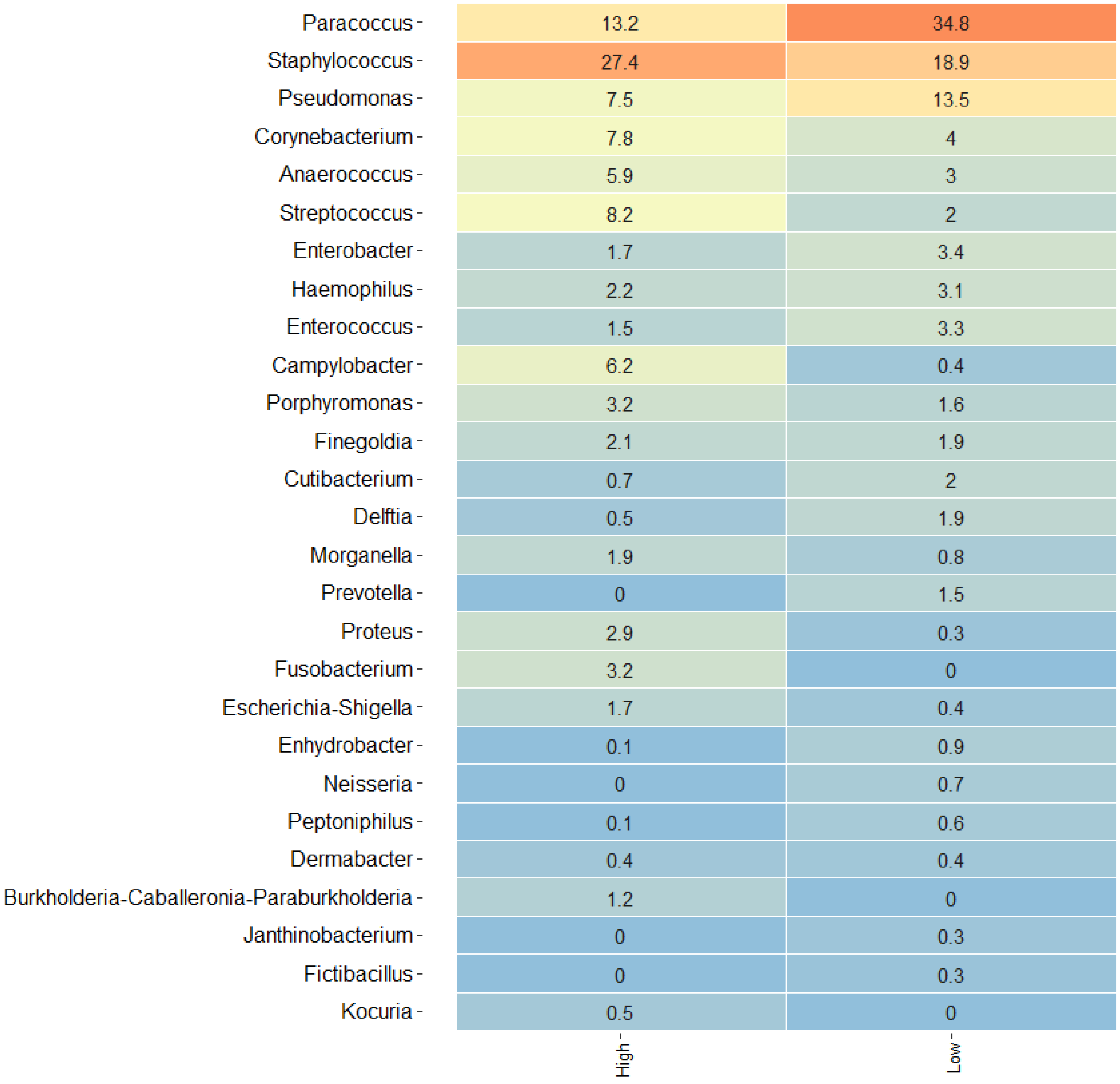

Paracoccus - 13.2 34.8
Staphylococcus - 27.4 18.9
Pseudomonas - 7.5 13.5
Corynebacterium - 7.8 4
Anaerococcus - 5.9 3
Streptococcus - 8.2 2
Enterobacter - 1.7 3.4
Haemophilus - 2.2 3.1
Enterococcus - 1.5 3.3
Campylobacter - 6.2 0.4
Porphyromonas - 3.2 1.6
Finegoldia - 2.1 1.9
Cutibacterium - 0.7 2
Delftia - 0.5 1.9
Morganella - 1.9 0.8
Prevotella - 0 1.5
Proteus - 2.9 0.3
Fusobacterium - 3.2 0
Escherichia-Shigella - 1.7 0.4
Enhydrobacter - 0.1 0.9
Neisseria - 0 0.7
Peptoniphilus - 0.1 0.6
Dermabacter - 0.4 0.4
Burkholderia-Caballeronia-Paraburkholderia - 1.2 0
Janthinobacterium - 0 0.3
Fictibacillus - 0 0.3
Kocuria - 0.5 0
High -
Low -
